# In this month’s *Bulletin*

**DOI:** 10.2471/BLT.15.000215

**Published:** 2015-02-01

In the editorial section, Steven J Hoffman et al. (66) argue for a binding international legal framework to address antimicrobial resistance. Steffan Crausaz (67) describes how New Zealand introduced competition among suppliers for medicines to treat rare disorders.

Fiona Fleck reports (70‒71) on the difficulties of testing treatments for Ebola virus disease. In an interview, Cheikh Niang (72‒73) explains why testing new vaccines, treatments and diagnostics in this context requires more than just a biomedical approach.

## Cambodia

## Too little, too late

Lily D Yan et al. (84‒92) document presenting complaints, treatment and outcomes of adults seeking emergency care in public hospitals.

## Zambia

## Bringing health care to prisons

Katie R Maggard et al. (93‒101) screen for tuberculosis and human immunodeficiency virus in prisoners and staff.

## Mozambique

## Changing the way tuberculosis is diagnosed

James Cowan et al. (125‒130) report challenges in rolling out rapid tests.

## India

## Missing the mark on rabies control

Syed Shahid Abbas & Manish Kakkar (131‒132) list discrepancies between evidence of what works and how control programmes are designed.

## Global

## More accurate doses; less drug resistance

Daniel J Hayes et al. (74‒83) build a model to improve age-based dosing of antimalarials.

## The high costs of health care

Beverley M Essue et al. (102‒112) review the evidence on ways to decrease household expenditure.

## Banks of biological specimens

Haidan Chen & Tikki Pang (113‒117) call for global governance of these collections.

## Beyond gross domestic product per capita

Elliot Marseille et al. (118‒124) discuss alternative approaches to setting cost‒effectiveness thresholds.

